# Two-port Endoscopic Surgery for Carpal Tunnel Syndrome – A Prospective Cohort Study

**DOI:** 10.5704/MOJ.2207.007

**Published:** 2022-07

**Authors:** TT Nguyen, K Duong, SQ Tran, KD Dang, HHV Ly, BTT Nguyen

**Affiliations:** 1 Department of Orthopaedics, Can Tho University of Medicine and Pharmacy, Can Tho, Vietnam; 2 Center for Trauma and Orthopaedics, Can Tho Center General Hospital, Can Tho, Vietnam; 3 Department of Pharmacy, Can Tho University of Medicine and Pharmacy, Can Tho, Vietnam

**Keywords:** carpal tunnel syndrome, endoscopic carpal tunnel release, transverse carpal ligament, thenar atrophy, repetitive wrist use

## Abstract

**Introduction:**

Carpal tunnel syndrome (CTS) is one of the most common peripheral neuropathies affecting patients' life. Performing endoscopic carpal tunnel release is now a new technique that is being gradually applied in Vietnam. This paper seeks to investigate the effectiveness of Chow’s method for CTS treatment.

**Materials and methods:**

This is a prospective cohort study involving seventy-seven patients with CTS who underwent Chow’s endoscopic method at our hospital from March 2019 to January 2020. The Boston Carpal Tunnel Questionnaire and electromyography (EMG) were used primarily to evaluate surgical decompression pre-operatively, one week, three weeks, three months, and six months after surgery. We also recorded incision length, pain at the scar, the improvement of symptoms and thenar atrophy and return-to-work time after surgery.

**Results:**

A total of 85.7% of the patients were women. A moderate severity of EMG was seen in 64.9% of cases. Six-month post-operative functional status scale (FSS) (1.05±0.1) and symptom severity scale (SSS) (1.05±0.1) showed significant improvement when compared with preoperative FSS (2.8±0.5) and SSS (3.2±0.5). Post-operative EMG showed the distal sensory latency (DSL) and distal motor latency (DML) had returned to the norm in 88% and 89.3%, respectively. The average incision length was 12.1±1.2mm. Six months after surgery, numbness and hand pain had resolved in 97.4%, a painless scar was seen in 94.7%, but full recovery of thenar atrophy was only seen in 9.1%. Patients could get back to work after 10.2±2.4 days.

**Conclusion:**

Chow’s endoscopic carpal tunnel release is a safe and effective procedure for patients suffering from carpal tunnel syndrome that showed promising outcomes on clinical symptoms and functions on EMG with minimal pain and scarring, and early return to work.

## Introduction

Carpal tunnel syndrome (CTS) is caused by compression of the median nerve in the carpal tunnel. It is one of the most common peripheral neuropathy seen in about 1% - 10% of the population^1^ and more common in women aged between 40-50 years^2^. It is often caused by incorrect wrist posture when working continuously in a fixed or flexed position for a long time^3^. CTS's most frequent symptoms include numbness and paraesthesia in the part of the hand distributed by the median nerve^4,5^. Conservative treatment is often indicated for patients who suffer mild symptoms or brief duration of illness. However, applying conservative treatment for a long time or having many severe symptoms can increase median nerve damage, leading to impairment and disability. Therefore, surgery should be considered timely for appropriate patients^6,7^.

The transverse carpal ligament release was described to treat post-traumatic median nerve compression in 1933 by Learmonth and treat non-traumatic median nerve compression in 1946 by Cannon and Love. Open carpal tunnel release has been the surgical standard in treating carpal tunnel syndrome. The endoscopic method was first introduced in 1987 by a Japanese orthopaedic surgeon. In 1989, Chow reported using two incisions to transect the transverse carpal ligament using an endoscope^8^. Endoscopic carpal tunnel release had many advantages over open surgery, such as a small scar, high aesthetics, less pain after surgery, and helping patients return to work earlier^6^.

There are many reports of carpal tunnel syndrome and its treatment results with transverse ligamentectomy worldwide. However, there are very few reports about endoscopic surgery in Vietnam. We conducted this study to investigate the clinical characteristics and EMG of CTS patients and evaluate the results of endoscopic carpal tunnel release (ECTR) at Can Tho Central General Hospital.

## Materials and Methods

We performed a prospective cohort study. For seven years from 2001 to 2007, Lam CH *et al* studied endoscopic carpal tunnel release in a Chinese population to assess its outcome^9^. They found that 68% of the patient’s hands had improved symptoms after surgery. Using the formula for estimating a proportion, we calculated the sample size of 70 was needed for our study. Seventy-seven patients were recruited in our study. After clinical examination, an EMG was done and the severity of CTS was classified according to Padua (1997)^10^. Patients with moderate or severe CTS, moderate CTS with failure of non-operative treatment or CTS with thenar atrophy were included for surgery. They underwent endoscopic carpal tunnel release at Can Tho Central General Hospital from March 2019 to January 2020.

We excluded patients with a history of other compression neuropathies, wrist injuries, or surgeries. We also excluded pregnant patients, patients with recurrent CTS, and patients who did not attend follow-up examination, could not be contacted or did not agree to join the study.

General characteristics including age, gender, occupation, CTS hand, illness duration, risk factors and associated conditions and previous conservative treatments were documented. Hand pain and numbness were the most common reasons patients came to us. Before surgery, all patients’ clinical characteristics were checked. We studied hand pain and numbness severity and looked for thenar muscle wasting. We also assessed the ability of thumb opposition, handgrip, Phalen’s manoeuvre, and Tinel’s sign. Electromyographic was indicated not only for detecting CTS but also for observing the response after surgery.

**Fig 1:**
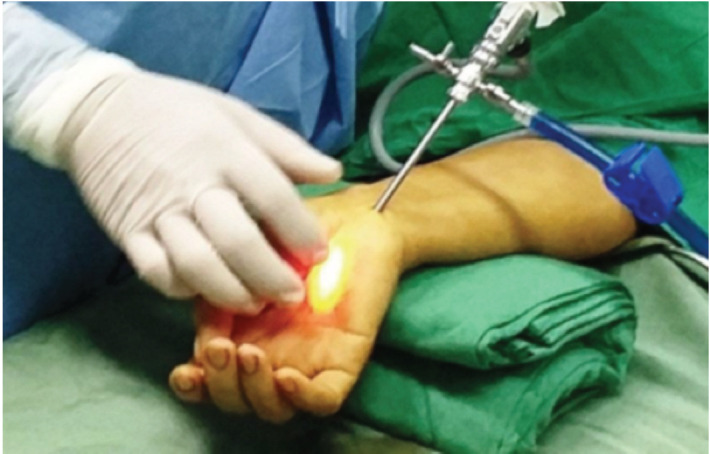
Determination of the second incision.

**Fig 2:**
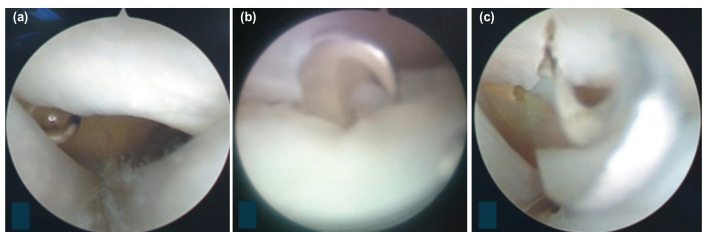
In operation: (a) Transverse carpal ligament before transection, (b) being transected using a reverse cutting blade, and (c) after transection.

All surgery was performed using Chow’s method by a well-trained orthopaedist in the Center for Trauma and Orthopaedics of Can Tho Central General Hospital. Patients were provided with pre-operative explanation regarding treatment methods, the ability to recover, and the complications during and after the surgery. Surgical instruments included an endoscope, a camera apparatus, a slotted cannula, and a reverse cutting blade. The patients were placed in a supine position. Under adequate local or general anaesthesia, we applied a tourniquet at the proximal arm and inflated it to about 250mmHg. We approached the carpal tunnel with an incision over the 1^st^, bracelet line, between the palmaris longus tendon and the flexor carpi ulnaris tendon. We were also cautious not to cut the palmar cutaneous branch. The endoscope and a light cord under the carpal transverse ligament were inserted to make a tunnel. Using the light to determine the second incision 3-4cm distally (Fig. 1), a slotted cannula was inserted through the 1st portal, and the endoscope was moved to the 2^nd^ portal. When the transverse carpal ligament was observed (Fig. 2a), we used the reverse blade to cut it from a distal to a proximal position (Fig. 2b). The transverse carpal ligament was rechecked to ensure totally transection (Fig. 2c). The tourniquet was deflated, and bleeding controlled. The two incisions were closed with single stitches at the end of the process.

We examined the patients four times after the operation: at one week, four weeks, three months and six months. We used the Boston Carpal Tunnel Questionnaire to evaluate surgical outcomes, including symptom severity and functional status before and after the intervention. We also observed the change in post-operative EMG, which was a valuable tool to re-evaluate the improvement after surgical decompression. Other features that we investigated were the remission of thenar atrophy, incision length and pain at the scar, and the time needed for patients to get back to work.

This study was approved by the Medical Ethics Councils of Can Tho University of Medicine and Pharmacy with the decision number 401/QD-DHYDCT signed on February 20^th^, 2019. Participants were informed of the purpose and procedure of the study and then they voluntarily signed a consent form.

Data were analysed by Statistical Package for Social Sciences (SPSS) version 22.0 [IBM Corp., New York, the United States of America] for Windows. The χ^2^ was used to investigate the relationship between the rates in the case of two qualitative variables. ANOVA test was used to find the correlation between mean values in the same subgroup. The T-Test was used to compare the mean values at two timepoints in the same group. Correlation coefficient r determines the degree of correlation in the correlation relationship between the variables. P-values <0.05 were considered statistically significant.

## Results

Table I showed the demographics of the patients. There were 77 patients with carpal tunnel syndrome, of which women made up 85.7%. The average age was 50.6±12.4. The disease duration was 1-3 years in 57.1%, many of them were treated conservatively with anti-inflammatory and analgesic medication (74%).

**Table I:** General Characteristics

	N	%
Age		
Under 40	16	20.8
40-50	23	29.9
51-60	25	32.4
Mean: 50.6 ± 12.4		
Gender		
Male	11	14.3
Female	66	8.7
Occupation		
Farmer	23	29.9
Worker	8	10.4
Officer	8	10.4
Salesclerk	7	9.1
Homemaker	23	29.9
Retired	8	10.4
Duration of illness		
Under 1 year	26	33.8
1 - 3 years	44	57.1
Over 3 years	7	9.1
Risk factors and associated conditions		
Menopause	35	45.5
Repetitive wrist use (6 – 8h)	70	90.9
Arthritis	35	45.5
Diabetes mellitus	6	7.8
Trigger finger	20	26.0
Obesity	12	15.6
Previous treatment		
Oral anti-inflammatory and pain relief	57	74
Oral anti-inflammatory and pain relief + Corticosteroid injection	20	26
Total	77	100

Pre-operative characteristics were described in Table II. Most of the patients complained about hand numbness when driving or working (94.8%), night-time numbness (87%), repetitive hand movement affected most at the carpal tunnel (90.9%). We recorded positive Phalen’s manoeuvre at 81.8% and Tinel’s sign at 71.4%. Thenar atrophy was seen in 24.7% of cases. The mean SSS (3.2±0.5) was higher than FSS (2.8±0.5). According to Padua evaluating system, 64.9% of patients were classified as moderate CTS while 10.4% met severe CTS criteria (Table III).

**Table II:** Pre-operative characteristics

	N		%
Site			
Left	37		48.1
Right	40		51.9
Symptoms			
Night-time numbness	67		87.0
Paresthesia in median nerve distribution	52		67.5
Numbness when driving or working	73		94.8
Hand pain	60		77.9
Signs			
Thenar atrophy	19		24.7
Loss of grip strength	24		31.2
Wrist vibration	10		13.0
Loss of manual dexterity	19		24.7
Weakness in thumb opposition	27		35.1
Phalen’s maneuver	63		81.8
Tinel’s sign	55		71.4
Boston Carpal Tunnel Score	Pre-op SSS	Pre-op FSS	T-Test
Mean	3.2±0.5	2.8±0.5	P<0.001
Total	77		100

**Table III:** Padua electromyography evaluation system

	N	%
Mild	50	64.9
Moderate	19	24.7
Severe	8	10.4
Total	77	100

The Boston Questionnaire reported FSS and SSS were improved significantly after surgery. It is shown in Table IV compared to the pre-operative score (p<0.05). Mean SSS decreased from 2.6±0.4 at 1-week post-op to 1.05±0.1 at 6-months post-op; mean FSS also decreased from 2.5±0.4 at 1-week post-op to 1.05±0.1 at 6-months post-op.

**Table IV:** Post-operative Boston FSS and SSS improvements; post-operative symptoms and thenar atrophy improvements

		1-week post-op	4 weeks post-op	3 months post-op	6 months post-op
Post-operative Boston score improvements (n=77)	Mean SSS	2.6±0.4	1.9±0.4	1.3±0.2	1.05±0.1
	Mean FSS	2.5±0.4	1.7±0.3	1.2±0.2	1.05±0.1
Symptom improvements (n=77)	No change	0	2.6	2.6	2.6
	Decreased	90.9	9.1	0	0
	Fully recovered	9.1	83.3	97.4	97.4
Thenar atrophy improvement (n=19)	No change	-	24.7	18.2	5.2
	Improved	-	0	6.5	10.4
	Fully recovered	-	0	0	9.1

The electromyographic results (Table V), which showed no signal on DSL and DML measurements accounted for 35.1% and 10.4%, respectively; on the contrary, prolonged DSL and DML were seen in 64.8% and 89.6%, respectively. In patients where DSL and DML were not recorded, the corresponding DSLd and DMLd could not be calculated. At 3-months after surgery, DSL, DML, DSLd, and DMLd on the electromyography returned to the norm in 29.3%, 54.7%, 45.3%, and 1.3%, respectively. Six-months after surgery these rates were all over 80%.

**Table V:** Electromyography improvements

EMG measurements	Pre-operation (n=77)		3 months Normal post-op (n=75)		6 months post-Normal	
	No signal	Prolonged	No signal	Prolonged	No signal	Prolonged
DSL	35.1%	64.9%	29.3%	70.7%	88%	12%
DML	10.4%	89.6%	54.7%	45.3%	89.3%	10.7%
DSLd	35.1%	64.9%	45.3%	54.7%	82.7%	17.3%
DMLd	10.4%	89.6%	1.3%	98.7%	82.7%	17.3%

At 6-months post-surgery, numbness and hand pain resolved in 97.4%. However, only 7 (9.1%) patients from 19 (24.7%) with thenar atrophy fully recovered. We found that the average length of the incision was 12.1±1.2mm. The shortest was 10mm, and the longest was 16mm. A total of 59.7% of patients had an incision length of 12 to 14mm. At 3-month post-operatively, 22.7% of the patients still had minimal pain at the surgical scar, which decreased to 5.3% after 6-months. This change was statistically significant at p<0.001. There was a positive correlation between incision length and return-to-work time. This correlation was statistically significant (p<0.001) (Table VI).

**Table VI:** Incision length, back-to-work time, and their correlation

	Correlation		Mean	Correlation Coefficient	Pearson test
Incision length (n=77)	<12mm	33.8%	12.1±1.2	r=0.4	p<0.001
	12-14mm	59.7%			
	>14mm	6.5%			
Back-to-work time (n=75)	< 7 days	6.7%	10.2±2.4		
	7-9 days	25.3%			
	10-12 days	50.7%			
	> 12 days	17.3%			

Two cases did not shown any improvement in their symptoms; CTS recurred in patients with prolonged wrist immobility and not enough physical therapy. There was no median nerve or tendon injury during the operation.

## Discussion

This study involved 77 patients, 85.7% of whom were women. The 40-60-year-olds was most affected, accounting for 62.3%. The moderate and severe CTS on EMG was seen in 64.9% and 10.4%, respectively. Boston Carpal Tunnel Questionnaire and post-operative electromyographic were primarily used to assess surgical decompression. Six months after post-operative FSS (1.05±0.1) and SSS (1.05±0.1) showed a significant improvement in comparison with preoperative FSS (2.8±0.5) and SSS (3.2±0.5). Post-operative EMG showed the DSL and DML returned to the norm in 88% and 89.3%, respectively. The average incision length was 12.1±1.2mm. At 6-months after surgery, numbness and hand pain had resolved in 97.4% while 94.7% had pain-free scars. However, full recovery of thenar atrophy was only seen in 9.1%. Patients could get back to work after 10.2±2.4 days.

In this study, 85.7% of patients were women in 77 patients. This has also been reported in many other studies, although with different rates. CTS is common in middle-aged women, mostly due to changes in the hormone, oestrogen^3^. Another causative factor is that the carpal tunnel area in women is smaller than in men as women's hands are smaller than men's^1,3^. The mean age of the patients was 50.6±12.4; this was consistent with studies of many other authors^11,12^. In general, the common age for carpal tunnel syndrome was from 40 to 60 years old and related to the decrease of elasticity of the carpal transverse ligament. The most common risk factor was repetitive wrist movement 6-8 hours/day (90.9%), more common in patients doing housework (29.9%) or farmers (29.9%).

Repetitive wrist movements and wrist vibration increases pressure on components in the carpal tunnel and causes this syndrome, respectively, comparable with previous estimates^13-15^. Most of the patients in our study had symptoms for three months or more. A total of 57.1% of the patients had symptoms for 1 to 3 years, while 33.8% had symptoms between 6-month-to-1-year. The above results were also relatively consistent with the results of some other studies. Patients often noted the silent progression of the disease, with slow onset and patient tolerance. A total of 74% of the patients had previously received anti-inflammatory and analgesic therapy at the time of registration. Surgical intervention should be indicated for patients who fail with conservative treatment.

The most common symptom was hand numbness at night (87%) or when driving or working 94.8%. Numbness in the median nerve distribution was seen in 67.5% of patients, while hand pain was 22.1%. Other studies have also noted that the most common symptom is numbness in the median nerve distribution. However, our study results differed from other studies in the degree of the disease^16^. CTS signs included weakness of thumb opposition (35.1%), loss of grip strength (31.2%), loss of manual dexterity (24.7%), wrist vibration (13%), and thenar atrophy (24.7%). The differences in many studies could be explained by different sampling criteria and different sample sizes^5,17^. Positive Phalen’s manoeuvre was seen in 81.8% and Tinel’s sign in 71.4%, respectively, consistent with the research results of many other authors. Although the rates vary, the authors believed this was one of the most valuable clinical signs in diagnosing carpal tunnel syndrome^18-21^. However, the diagnosis of these signs depended a lot on the surgeon’s skills. According to some literature, the more severe the disease and later the stage, the lower the positive rate of these signs^18,22,23^.

The Boston Questionnaire was a reliable tool for evaluating the results of carpal tunnel decompression. The average preoperative Symptom Severity Score was 3.2±0.5, higher than the Functional Status Score of 2.8±0.5 (p<0.001). Our patients had an average of 64.9% of the neurological damage on EMG when there was only sensory conduction damage to the median nerve and no motor conduction damage. In contrast, at the level of moderate and severe EMG (24.7% and 10.4%, respectively), the effect on motor conduction causes hand dysfunction. With the results, the mean preoperative FSS correlated positively with the Padua evaluation on electromyography. The more severe the disease, the higher the mean pre-operative FSS score (p<0.001). With FSS and SSS, at the time of 6 months post-op, the score had improved to 1.05±0.1 and 1.05±0.1, respectively, which was statistically significant with p<0.05. Other studies by Gurpinar or Gumustas *et al* also recorded a marked change in average Boston FSS and SSS before and after surgery^12,17^. The symptom recovery was faster and could be seen after one to four weeks, while the functional recovery was more gradual, lasting from three to six months. Other studies also showed a positive correlation between the average Boston FSS and SSS and the electromechanical resolution of the median nerve^24,25^.

There was a statistically significant improvement of EMG after surgery (p<0.001). The distal motor latency the distal sensory latency, and their differences returned to the norm after 6-months of surgery in over 80% of patients. These results were consistent with the research results of other authors^26^.

One week after surgery, all patients expressed improvement in symptoms. At 4-weeks post-operatively, 83.3% of patients had full resolution. There were 2 cases (2.6%) in which there were no improvements of the symptoms; CTS recurred in patients with wrist immobility and poor physical therapy after 1 week of operation. At 6 months post-surgery, the rate of patients whose symptoms had fully recovered increased to 97.4%. Our results were somewhat similar to other studies^5^. In 24.7% of patients with thenar atrophy before surgery, there was no change at 4 weeks after surgery. However, at 3 months and 6 months after surgery, 6.5% and 9.1% of patients fully recovered from thenar atrophy, respectively, while partial recovery rate was 10.4%, and no change was seen in 5.2%. Our results were similar to other studies. Thenar atrophy was considered a complication of CTS due to long-term compression of the median nerve. The longer the compression time on the median nerve, the more degenerative the axon and the greater the degree of muscle atrophy seen. After surgery, the recovery of thenar atrophy could be very slow, and in some cases, it may not be reversed^23,24^.

With Chow’s method, we were also interested in the length of the incision, recording an average of 12.1±1.2mm, of which the shortest was 10mm, and the longest was 16mm. The authors Gurpinar or Heidarian *et al* also noted that the endoscopic incision is always smaller than the conventional surgery^12,27^. A total of 22.7% of patients presented with pain at the surgical scar at 3 months post-op, and just 5.3% of them still had pain at the scar at 6 months post-op. Gurpinar found that at the time of 3-month post-op, pain at the scar between the endoscopic release group and open release group was seen in 7.4% and 16%, respectively, and pain-free in the endoscopic release group at 1-year post-op^12^. The average back-to-work time was 10.2±2.4 days. We observed a positive correlation between back-to-work time and incision length (r=0.4, p<0.001) and similarities with other studies^28-30^. Endoscopic surgery helped limit wrist soft tissue damage, short skin incisions, smaller surgical scar with minimal pain, and early return to work or daily activities compared to conventional surgery.

Complications from endoscopic carpal tunnel release are injury to nerves or tendons^31^. Nerve or tendon injuries could affect the primary outcome and were seen as a procedure failure. In our study, we did not record those injuries. We carefully performed this method with every hand we operated on, from approaching the tunnel to cutting the transverse carpal ligament under good visualisation.

It is plausible that several limitations might have influenced the results. The number of patients was just 77 patients, showing limited power. We did not completely assess the severity of the signs, which were needed to use specific tools that would help predict and objectively evaluate the treatment results. On a few occasions, we met some difficulties in manipulating instruments that unintentionally made the incisions larger and affected the result of incision length.

Endoscopic carpal tunnel release has become a more common procedure for treating carpal tunnel syndrome worldwide. Previous studies have demonstrated that ECTR helps patients reduce pain and return to work earlier. Besides, ECTR was adduced to be more effective in restoring hand strength and fewer complications than open release surgery^7,32^. In Vietnam, endoscopic carpal tunnel release is still new among orthopaedic surgeons so far, and less work has been done to evaluate its outcomes. This study demonstrated the endoscopic method's effectiveness and encouraged practitioners to apply it in treating CTS.

## Conclusions

Chow’s endoscopic carpal tunnel release is a safe and effective procedure for patients suffering from carpal tunnel syndrome that showed promising outcomes on clinical symptoms and functions, on EMG with minimal pain and scarring, and earlier return to work.
